# First-Principles Study on the Stabilities, Electronic and Optical Properties of Ge_x_Sn_1-x_Se Alloys

**DOI:** 10.3390/nano8110876

**Published:** 2018-10-25

**Authors:** Qi Qian, Lei Peng, Yu Cui, Liping Sun, Jinyan Du, Yucheng Huang

**Affiliations:** College of Chemistry and Material Science, The Key Laboratory of Functional Molecular Solids, Ministry of Education, Anhui Laboratory of Molecule-Based Materials, Anhui Normal University, Wuhu 241000, China; qianq@ahnu.edu.cn (Q.Q.); penglei@ahnu.edu.cn (L.P.); cuiyu@ahnu.edu.cn (Y.C.); sunlp@ahnu.edu.cn (L.S.); dujinyn@mail.ahnu.edu.cn (J.D.)

**Keywords:** GeSe monolayer, SnSe monolayer, alloy, first-principles, electronic property, optical property

## Abstract

We systematically study, by using first-principles calculations, stabilities, electronic properties, and optical properties of Ge_x_Sn_1-x_Se alloy made of SnSe and GeSe monolayers with different Ge concentrations x = 0.0, 0.25, 0.5, 0.75, and 1.0. Our results show that the critical solubility temperature of the alloy is around 580 K. With the increase of Ge concentration, band gap of the alloy increases nonlinearly and ranges from 0.92 to 1.13 eV at the PBE level and 1.39 to 1.59 eV at the HSE06 level. When the Ge concentration x is more than 0.5, the alloy changes into a direct bandgap semiconductor; the band gap ranges from 1.06 to 1.13 eV at the PBE level and 1.50 to 1.59 eV at the HSE06 level, which falls within the range of the optimum band gap for solar cells. Further optical calculations verify that, through alloying, the optical properties can be improved by subtle controlling the compositions. Since Ge_x_Sn_1-x_Se alloys with different compositions have been successfully fabricated in experiments, we hope these insights will contribute to the future application in optoelectronics.

## 1. Introduction

Since the emergence of grapheme [1], two-dimensional (2D) materials have attracted intense attention in the scientific community due to richness of the physical properties [2]. It is known that although graphene has high conductivities, the feature of having a zero band gap greatly restricts its application in the semiconductor industry [3]. As important supplements of graphene [4,5], 2D layered materials such as transition-metal dichalcogenides (TMDs) [6], black phosphorene (BP) [7], hexagonal boron nitride (h-BN) [8], metal carbides and carbonitrides (MXenes) [9], and monoelemental arsenene, antimonene [10], bismuthene [11], silicone [12], germanene [13], tellurene [14], etc., have been experimentally manufactured or theoretically predicted which exhibit unique electronic and optical properties for broad applications at the nanoscales. Versatile and complementary properties of these 2D materials can meet a large variety of requirements for potential application. A typical example is MoS_2_ [15,16,17] that has attracted tremendous attention among the TMDs materials. While it has a large intrinsic band gap of 1.8 eV [18], the reported mobilities are only in the range of 0.5–3 cm^2^ V^−1^ s^−1^, which are too low for practical application in electronic devices [4]. Recently, the booming 2D materials of BP seems to make up the gap between graphene and MoS_2_ because it has a tunable direct band gap from 0.3 eV of the bulk to 1.5 eV of the monolayer [19]. The resulted high theoretical mobility [7,19], excellent near-infrared properties [20] as well as high photoelectric conversion capacity [21] endow its widely potential application in the field of electronic and optical devices. However, the stability of phosphorene in air and water needs to be enhanced. Thus, it seems that the single-component materials always have some disadvantages that greatly affect their widespread applications. 

Alloyed 2D semiconductors can display compositionally tunable properties, which distinct from both their bulk alloys and binary alloy end-members. Through forming heterojunctions, the limitation of single-component 2D materials is expected to be broken through. Abundant researches on different kinds of the heterostructures, such as van der Wall (vdW) heterostrutures, lateral (in-plane) heterostructures and solid solution heterostrutures, have confirmed that the properties of single materials can be tailored by mixing with the second components, which provides a feasible way to improving the electronic, optoelectronic, as well as the catalytic properties of nanostructures [19,22,23,24,25]. For examples, the band gap of graphene is considerably open when vdW heterostructure of graphene/g-C_3_N_4_ bilayer is formed [26]. Likewise, through forming MoS_2_-WS_2_ heterostructure, the carrier mobility is greatly enhanced to 65 cm^2^ V^−1^ s^−1^ [27]. Moreover, a facile and general method to passivate thin BP flakes with large-area high-quality monolayer h-BN sheets grown by the chemical vapor deposition (CVD) method was developed to preserve atomic layered BP flakes from degradation [28]. 

Recently, our group [29] has studied the electronic and optical properties of SnSe_2x_S_2(1-x)_ anion alloy through first-principles calculations. It was found that the band gap is not confined in a certain value, but varies in the range of the band gap of SnSe_2_ to that of SnS_2_, depending on the ratio of SnSe_2_ and SnS_2_. The adsorption strength is enhanced in the visible spectral region after alloying. Moreover, the alloys are predicted to be stable and would be favorably fabricated from the calculation results of phase diagram. Shortly after that, Wang et al. [30] have experimentally prepared 2D SnSe_2(1-x)_S_2x_ alloys with five different S compositions (x = 0, 0.25, 0.5, 0.75, and 1) by the chemical vapor transport (CVT) method. Different from the independent SnSe_2_ or SnS_2_ monolayer, carrier mobility of SnSeS field-effect transistor can be obviously increased by light illumination of 532 nm laser, indicative of the potential application as a phototransistor. Besides, different cation alloys, such as Mo_1-x_W_x_S_2_, Mo_1-x_W_x_Se_2_, etc., have also been successfully synthesized which exhibit different functions with respect to their end-members [31,32,33]. Therefore, novel properties can be obtained by alloying different 2D materials. 

Due to high stability, earth abundance and environmental sustainability, 2D group IVA monochalcogenides (MXs), i.e., GeS, SnS, GeSe and SnSe [34,35,36,37], which are isostructural to black phase of phosphorene, have attracted intense attention recently. Experimentally, solid solutions can be formed with complete solid solubility among these MXs because of their structural similarity. Jannise et al [38]. have synthesized ternary Sn_x_Ge_1−x_Se nanocrystals and exhibited adjustable components over the entire alloy range (0 ≤ x ≤ 1). Compositional tuning on the lattice parameters, band gaps, and morphologies have been demonstrated and the alloy formation mechanism were thereby proposed. Moreover, Fu et al [39]. have successfully prepared Ge-doped SnSe polycrystals by the zone-melting combined with hot-pressing methods, and found that Ge is not an ideal dopant for optimizing the thermoelectric properties of SnSe. Since experimental investigations on the composite system made by GeSe and SnSe have been successfully carried out, however, as far as we know, theoretical investigations related to this system have not been reported yet. 

In this contribution, stabilities, electronic structures and optical properties of single-layer Ge_x_Sn_1-x_Se alloys with different Ge concentrations are systematically examined on the basis of density functional calculations. The soluble temperature of the alloy, variations of the electronic and optical properties as well as the underlying reasons are given. The potential application in optoelectronics is proposed.

## 2. Computational Details

First-principles calculations were carried out on the basis of density functional theory (DFT), as implemented in the Vienna Ab-initio Simulation Package (VASP) program [40,41]. The electron-ion interactions are descried by the projector-augmented plane wave (PAW) method [42]. Perdew−Burke−Ernzerhof (PBE) functional [43,44] in generalized gradient approximation (GGA) was used to process the electron-exchange correlation interactions unless stated otherwise and a 450 eV energy cutoff for the plane-wave basis sets were used. Vacuum height is set to 20 Å along x-direction to avoid the interactions between two periodic repeating units. The convergence thresholds for energy and force were set to 10^−5^ eV and 0.02 eV/Å, respectively. The Brillouin zone was represented by a Monkhorst−Pack [45] special k-point mesh of 1 × 3 × 3 for geometry optimizations, whereas a larger grid of 1 × 5 × 5 was used for band structure computations.

## 3. Results and Discussion

### 3.1. Structures and Stabilities of Ge_x_Sn_1-x_Se Alloys

**Figure 1:**
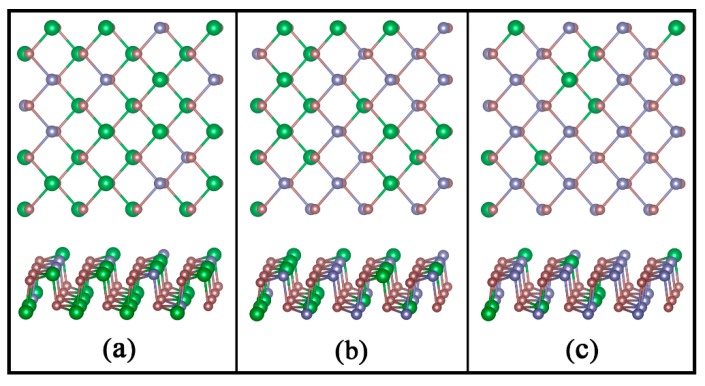
Top and Side Views of (**a**) Ge_8_Sn_24_Se_32_, (**b**) Ge_16_Sn_16_Se_32_ and (**c**) Ge_24_Sn_8_Se_32_ Monolayers with the Ge Concentration x of 0.25, 0.50 and 0.75, respectively.

To best approximate the random solid solution, special quasi-random structures (SQSs) with five different Ge compositions (x = 0, 0.25, 0.5, 0.75, and 1) of the Ge_x_Sn_1-x_Se alloys [46] were generated using the tool in the alloy theoretic automated toolkit (ATAT) [47]. Lattice parameters of each structure are then optimized, and a linear dependence on the composition is found, which is known as Vegard’s law [48]. In Figure 1, three typical alloys with the Ge concentration of 0.25, 0.50 and 0.75 are illustrated. Note that phase stability of the alloy is critical to the possibility of alloy formation. To verify the structural stabilities of these alloys, a thermodynamic calculation was performed as previously proposed [29]. 

First, the mixing enthalpy Δ*H_m_*(*x*) is calculated through subtracting the energy summation of pure GeSe (A) and SnSe (B) from the total energy of Ge_x_Sn_1-x_Se monolayer. That is,

$$\Delta H_{m}(x)=E(A_{x}B_{1-x})-\left[xE_{A}+(1-x)E_{B}\right]$$

**Figure 2:**
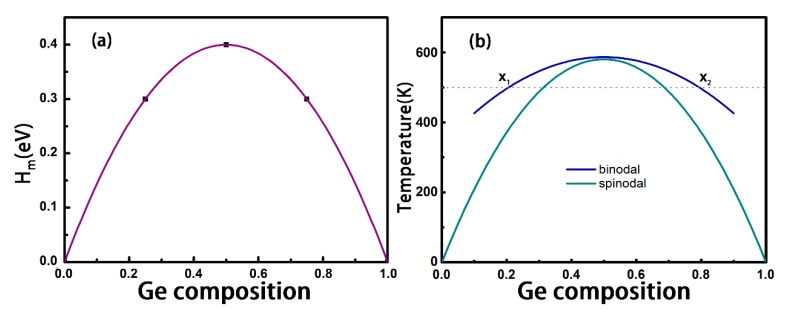
(**a**) Mixing Enthalpy and (**b**) Quasi-binary Phase Diagram as a Function of the Ge Content for the Ge_x_Sn_1-x_Se Alloys. Binodal and Spinodal Curves are Shown in Royal and Dark Cyan Lines in (**b**).

In Figure 2a, the calculated mixing enthalpies as a function of Ge concentrations are shown. It is found that the binary alloys always have positive mixing enthalpies, showing a phase separation tendency at low temperatures. Considering the contribution of the mixed entropy, the solubility of the alloy would be enhanced by increasing the temperature.

Here, the mixing entropy is calculated from a classical formula in the textbook [49]:

$$\Delta S_{m}\left(x\right)=-2\left[xlnx+\left(1-x\right)ln\left(1-x\right)\right]k_{B},$$

and the free energy of mixing is calculated corresponding to the following formula:

$$\Delta F_{m}\left(x\right)=\Delta H_{m}\left(x\right)-T\Delta S_{m}\left(x\right).$$

To obtain the critical temperature of alloy mutual solubility, a second-order polynomial based on the quasi-chemical model is used to describe the relationship between mixing enthalpies and Ge concentrations:

$$\Delta H_{m}\left(x\right)=\Omega x\left(1-x\right),$$

where Ω is the interaction parameter dependent on the material. Accordingly, the mixing Helmholtz free energy can be rewritten as

$$\Delta F_{m}\left(x\right)=\Omega x\left(1-x\right)+RT\left[xlnx+\left(1-x\right)ln\left(1-x\right)\right],$$

in which the value of Δ*F_m_* only relies on the Ge concentration *x*. Therefore, the binodal solubility curve and spinodal decomposition curve can be simply obtained by the formula of $\frac{\partial F_{m}}{\partial x}=0$ and $\frac{\partial^{2}F_{m}}{\partial x^{2}}=0$, respectively.

The obtained binary phase diagram is shown in Figure 2b, where we can see the binodal and spinodal curves meet at x = 0.5 at a critical temperature $T_{c}=\Omega /2R$. The symmetrical feature of the curve is consistent with the systems of Mo_1-x_T_x_S_2_ (T=W, Cr, and V) [17] and SnSe_2(1−x)_S_2x_ [29]. The calculated critical temperature is 580 K, which is an effortlessly realizable temperature in the laboratory, indicating that this kind of alloys can be favorably prepared. Note that if we count in the entropy contributed by lattice vibration, the solubility may be underestimated. In fact, the Sn_x_Ge_1-x_Se alloys have been successfully fabricated by Buckley et al., who heated precursor solutions to 500 K and held for 4.75 h with stirring [38]. 

As indicated in Figure 2b, when the temperature exceeds the critical temperature of 580 K, the alloys become stable in the whole range of composition in thermodynamics. At variance, when the temperature is lower than 580 K, for example, 500 K, the alloys can be formed only when the concentration of Ge is in the ranges of 0 < x < x1 and x2 < x < 1. In other words, when the concentration of Ge is within x1 < x < x2, the alloy becomes unstable and decomposes into components x1 and x2 phases.

On the other hand, the growth of Ge_x_Sn_1-x_Se alloys is believed as a cation exchange mechanism [38], which means that nucleation begins as SnSe (or a tin-rich selenide) and gradually incorporates Ge over the growth period. Thermodynamic driving force for the exchange can be evaluated from the calculations of substitution energy, which is defined as

$$E_{s}=E\left(doped\right)-E\left(pure\right)-n\mu_{Ge}+n\mu_{Sn}$$

where *E*(*doped*) and *E*(*pure*) are the total energies of the Ge-doped and pure SnSe supercells, μ_Ge_ and μ_Sn_ represent the chemical potentials of the Ge and Sn atoms, respectively. From this definition, the more negative of the *E_s_*, the easier of the alloy formation. The calculated substitution energies are listed in Table 1. These values are all considerably negative, suggesting that the Ge-Sn exchange mechanism accounts for the formation of alloys.

### 3.2. Electronic Properties of Ge_x_Sn_1-x_Se Alloys

**Figure 3:**
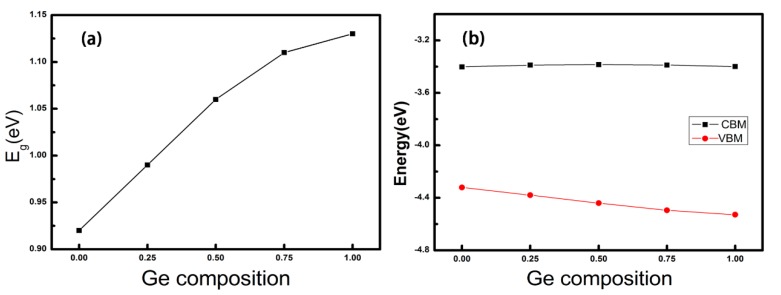
Calculated Band Gaps (**a**) and Band Edge Positions with Respect to Vacuum Energy Level (**b**) of Ge_x_Sn_1-x_Se Monolayers as a Function of the Ge Concentration.

**Figure 4:**
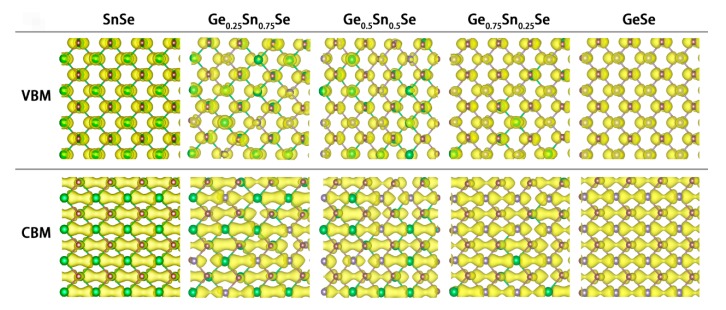
Partial Charge Densities at the VBM and CBM States for the Selected Alloys, where Green, Purple, and Grey Balls Represent Sn, Se and Ge Atoms, respectively.

Next, electronic properties of the alloys are investigated with the variation of Ge concentration x. As shown in Figure 3a, the band gaps of SnSe and GeSe are calculated to be 0.92 (indirect) and 1.13 eV (direct), respectively, which are well consistent with the results calculated at the same level, e.g., 0.96 and 1.18 eV [50]. It is observed that as the composition of the Ge_x_Sn_1-x_Se alloy becomes more Ge rich, the band gap gradually increases. Interestingly, this increase is approximately linear from x = 0 to x = 0.75, but displays a notable bending at x = 1. To analyze the reason for this observation, the band edge positions referenced to the vacuum energy levels of the alloys are given in Figure 3b. It can be seen that the positions of conduction band minimum (CBM) is almost invariable and linearly varied, while those of valence band maximum (VBM) decreases with less linearity, especially at the x = 1. Examining the partial charge densities of the alloys with the selected Ge concentrations shown in Figure 4, one can see that the densities at the CBM are always delocalized and evenly distributed. However, the densities at the VBM dominantly distribute around the Se atom, with a small distribution on Sn/Ge atoms, which seems thinner as the Ge concentration increases.

**Figure 5:**
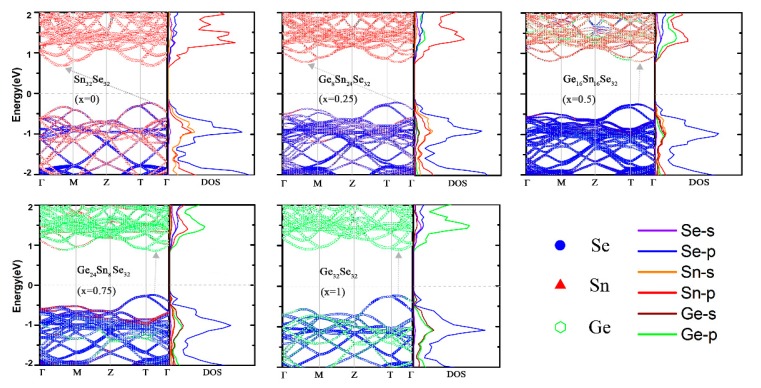
Atom-projected Band Structures and Density of States of the Different Ge_x_Sn_1-x_Se (x = 0.0, 0.25, 0.5, 0.75, and 1.0) alloys. The direct/indirect bandgap is indicated by the grey arrow.

Moreover, from Figure 3b, the increase of band gap with the Ge concentration is due to the decline of VBM edge position. To get further insight for this variation, the atom-projected band structures (atom contributions are indicated by different colors) and the density of states (DOS) are illustrated in Figure 5. As is seen, the atom contribution to the CBM changes from Sn to Ge, while the VBM is contributed by the hybridization of both Sn/Ge and Se atoms. This situation is different from the transition metal alloy systems, in which the VBM states are uniformly distributed among W and Mo d-orbitals [31]. Scrutinizing the DOS, for CBM, the main contribution of SnSe is from Sn-p orbital. With the concentration of Ge reaches 0.75, the main contribution changes to Ge-p orbital. While CBM states experience a bigger change of atom contribution, the energy distribution is nearly unchanged. This situation is because the formed anti-bonding π* orbital may suffer a less energy disturbation with the orbital composition change. In contrast, for the VBM, the contribution seems like from the Se-p orbit all the time, along with the considerable contribution from the hybridization of s- and p-orbitals of Sn/Ge. As the electronegativity difference between Ge (2.01) and Se (2.55) is smaller than that of between Sn (1.96) and Se, the resulting weaker bonding at the VBM with the Ge concentration increasing gives rise to the energy of VBM uplift.

Another interesting finding is the indirect to direct bandgap crossover occurs at the Ge concentration of 0.5. As is seen, SnSe is found to have an indirect bandgap, but with the Ge concentration increasing to 0.5, the alloy turns into a direct bandgap as the GeSe monolayer. While experimental investigations focusing on mono and few-layer SnSe and GeSe are scarce, the indirect/direct bandgap feature of SnSe/GeSe monolayers has been validated by previous reported theoretical results [50,51,52]. It is known that 2D materials with the direct bandgap is urgently needed as electrons can be directly excited from the VBM to the CBM by the incident light with an appropriate frequency without the aid of phonons, which leads to the potential application in the optical devices. Moreover, our Heyd–Scuseria–Ernzerhof (HSE06) [53] calculations give the band gaps of 1.39 and 1.59 eV for SnSe and GeSe monolayer, respectively, which are larger than those calculated at the PBE level whereas the band structure topologies are almost the same, with the main difference of CBM state upshift. Note that the HSE06 function used here is only to obtain more accurate band gap as the predicted band gaps of GeSe, SnSe bulks are very close to the experimental ones [50,52,54]. For example, the band gaps measured by Kim and Choi are 0.88 eV for SnSe single crystal and 1.10 eV for GeSe [54]. Theoretically, Shi and Kioupakis obtained the band gaps of 0.89 eV and 1.10 eV for SnSe and GeSe bulks at the HSE06 level, respectively [52]. Therefore, through forming alloys with x ≥ 0.5, on the one hand, the alloy becomes the direct bandgap semiconductor which facilitates the efficiency of light absorption; on the other hand, the alloy has the perfect band gap ranging from 1.50 to 1.59 eV, which is the most optimal value for the materials applied in solar cells. 

### 3.3. Optical Properties of Ge_x_Sn_1-x_Se Alloys

**Figure 6:**
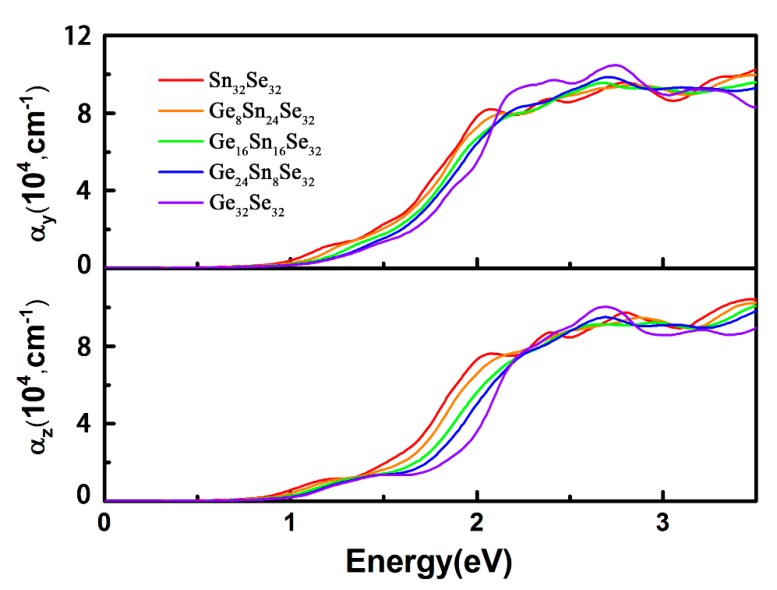
Optical Absorption Coefficients along the y and z Directions of the Ge_x_Sn_1-x_Se Alloys with Different Ge Compositions.

As described above, Ge_x_Sn_1-x_Se alloys have the band gap ranging from 0.92 to 1.13 eV at the PBE level or from 1.39 to 1.59 eV at the HSE06 level. Note that these variation scopes cover the main solar irradiation of the visible range. To further validate the potential applications on the photoelectronics and photovoltaics, optical absorption properties are conducted according to the Kramer-Kroing relationship [55]. The calculated optical absorption coefficients with the incident light polarization along the y and z directions are presented in Figure 6. First, an anisotropy feature is observed as the absorption coefficients along the y direction (armchair), which are significantly larger than those of z direction (zigzag). Second, with the Ge concentration increasing, the optical absorption edges along the y and z directions are blue-shifted, which is consistent with the order of the calculated band gaps. Third, the absorption coefficients reach around 10^5^ cm^−1^ when the photo energy is larger than 2.0 eV, especially the absorption strength is significantly enhanced through alloying. These observations notably verify that through alloying GeSe and SnSe, the optical properties can be improved by subtly controlling the compositions.

## 4. Conclusions

In summary, we have systematically studied the stabilities, electronic, and optical properties of Ge_x_Sn_1-x_Se alloys with the variation of Ge content through first-principles calculation. The structures of alloys were first generated by the tool of ATAT. Thermodynamic analysis show that the alloys are considerable stable, and the soluble temperature of GeSe and SnSe is estimated to be 580 K. The negative substitution energies of Ge replacing Sn in the SnSe monolayer verify the proposed cation exchange mechanism for the alloy growth. Electronic property calculations show that with the Ge concentration increasing, the band gap of alloys ranges from 1.39 to 1.59 eV at the HSE06 level. The non-linear increase of band gap, especially at the x = 1, is due to the uneven distribution of charge densities at the VBM states. Interestingly, when Ge concentration exceeds 0.5, on the one hand, the alloys have the direct bandgap which facilitates the efficiency of light absorption; on the other hand, the alloys have perfect band gaps in the range of 1.50–1.59 eV which falls into the most optimal scope for the application in solar cells. Optical calculations further verify that through alloying, the optical properties can be improved by subtly controlling the compositions. We believe that our work may shed light on the future applications of Ge_x_Sn_1-x_Se alloys in optoelectronics.

## Figures and Tables

**Table 1:** Substitution Energies (in eV) of the Ge_x_Sn_1-x_Se Alloys with Different Ge Contents.

**x**	**0.25**	**0.50**	**0.75**	**1.00**
***E_s_***	−3.77	−7.77	−11.87	−16.21
